# Infected Dentigerous Cyst of Maxillary Sinus Mimicking Intraoral Abscess in a Pediatric Patient: Management of Misdiagnosis and Inaccurate Treatment

**DOI:** 10.1155/2022/4852464

**Published:** 2022-05-23

**Authors:** Damian Chybicki, Alicja Popielarczyk, Wojciech Markowski, Gaja Torbicka, Anna Janas-Naze

**Affiliations:** Department of Oral Surgery, Central Clinical Hospital, Medical University of Lodz, Poland

## Abstract

The article presents the case of a 12-year-old male patient who was suffering from exacerbated (infected) dentigerous cyst of maxillary sinus, associated with impacted canine whose symptoms were deceptively similar to intraoral abscess. The first-aid treatment performed by general dental practitioner consisted of intraoral incision and resulted in oroantral fistula formation. Eventually, the patient's ailments were not resolved, and we had to deal with serious local complications and difficulties. The treatment was performed under general anesthesia in one-day surgery system and included enucleation of the lesion, excision, and closure of oroantral fistula. Dislocated canine was not removed, and its subsequent eruption was observed.

## 1. Introduction

Many different types of inflammatory lesions can be seen in the oral cavity of pediatric patients. Odontogenic cysts represent the most common form of cystic lesions affecting the maxillofacial region [1]. Dentigerous cysts (also known as follicular cysts) are the second most common form of developmental odontogenic cysts that occur over a relatively broad age range, most frequently in the second to fourth decade of life. These lesions are the second most common odontogenic cysts (after radicular cysts) and account for almost 24% of all true cysts of the jaws [2, 3]. It is recognized that the formation of this type of cysts results from fluid accumulation between residual enamel epithelium, which is attached to cementoenamel junction, and the crown of an unerupted tooth (most frequently mandibular third molars, maxillary canines, and mandibular premolars) [2, 4].

Development of dentigerous cysts, just like many other cystic lesions, is slow and asymptomatic. They are usually discovered accidentally during routine radiographic examination performed for any other reason. However, the size of these lesions may lead to facial asymmetry or displacement of adjacent teeth, which makes them indirectly noticeable even during extraoral examination [4, 5]. It needs to be highlighted that cysts may also be affected by a bacterial infection, which results in pain, edema and pus accumulation. They can be misdiagnosed with abscesses in cases when radiographic examination was not performed or was misinterpreted. Effects of such circumstances are presented in this paper.

## 2. Case Report

A 12-year-old male patient with no general diseases presented to Department of Oral Surgery, Medical University of Lodz due to pain and edema of the right part of his face. The patient was referred to our Department by a general dental practitioner who diagnosed an abscess and performed intraoral incision to evacuate a pus discharge and prescribed antibiotic (amoxicillin 500 mg every 12 h) but symptoms did not decrease for the next three days.

During extraoral examination edema and redness of the skin of the right infraorbital and buccal area were noticed. Intraoral examination revealed a bulge in the area of teeth 54-52 and an opening after incision with pus discharge outflow.

**Figure 1:**
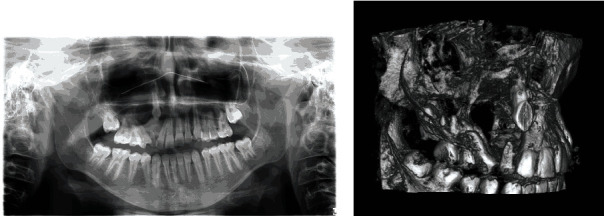
OPG (a) and CBCT 3D scan (b). A considerable size of the lesion and translocation of tooth 13 are visible (arrow).

Despite the patient's age, tooth 53 with slight mobility was still present in his oral cavity, so our diagnosis included a possible occurrence of infected dentigerous cyst. We performed an orthopantomogram (OPG) and CBCT (Cone-Beam Computed Tomography) which revealed a massive cyst-like lesion in the right maxillary sinus with translocation of tooth 13 (Figure 1) what confirmed our initial diagnosis. In the light of this evidence the previous dentist created an oroantral communication with his incision and after three days this opening became an oroantral fistula.

**Figure 2:**
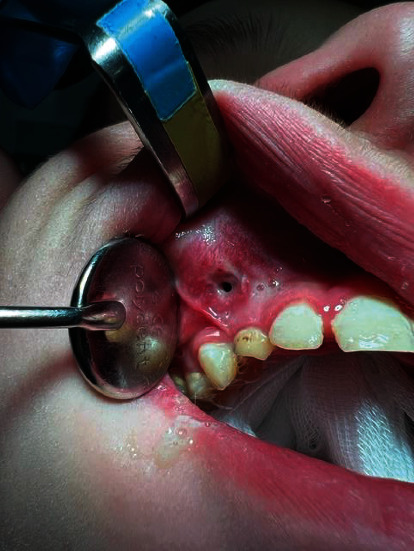
Intraoral view on operative region. Pus discharge is still present despite antimicrobial treatment.

We established a treatment plan which included daily rinsing of the cyst and maxillary sinus with 0.02% chlorhexidine solution for the next 7 days through the fistula and continuation of the antibiotic (amoxicillin 500 mg every 12 h) to eliminate the acute inflammation symptoms and subsequently excision of the fistula, extraction of tooth 53, and enucleation of the cyst under general anesthesia. Decision whether to save tooth 13 or not was planned to be made intraoperatively, depending on its exact position and mobility after enucleation. We presented the treatment plan to patient's parents, collected all the necessary consents, and gave all of the recommendations that must be followed before surgery (e.g., laboratory blood tests, i.e., complete blood count and APTT). Despite the antimicrobial treatment, pus discharge was still present (Figure 2). In our opinion, tooth 53 extraction should have been applied as first aid, which would have resulted in outflow of the discharge from infected cyst, as well as alleviation of acute inflammation symptoms. Subsequently, a surgery should have been scheduled with the flap designed without any deficiency in soft tissues (because fistula has to be excised) and without shallowing the vestibule after suturing as its result.

**Figure 3:**
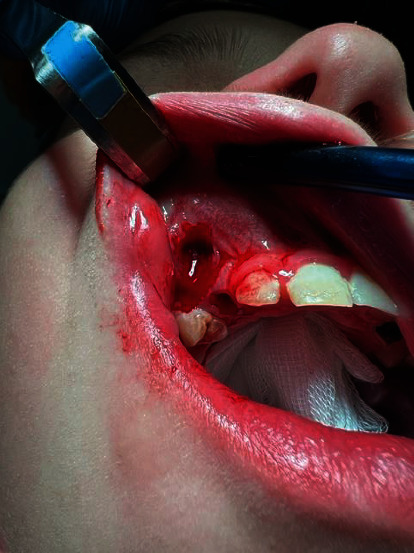
Tooth 53 is removed and oroantral fistula is excised.

**Figure 4:**
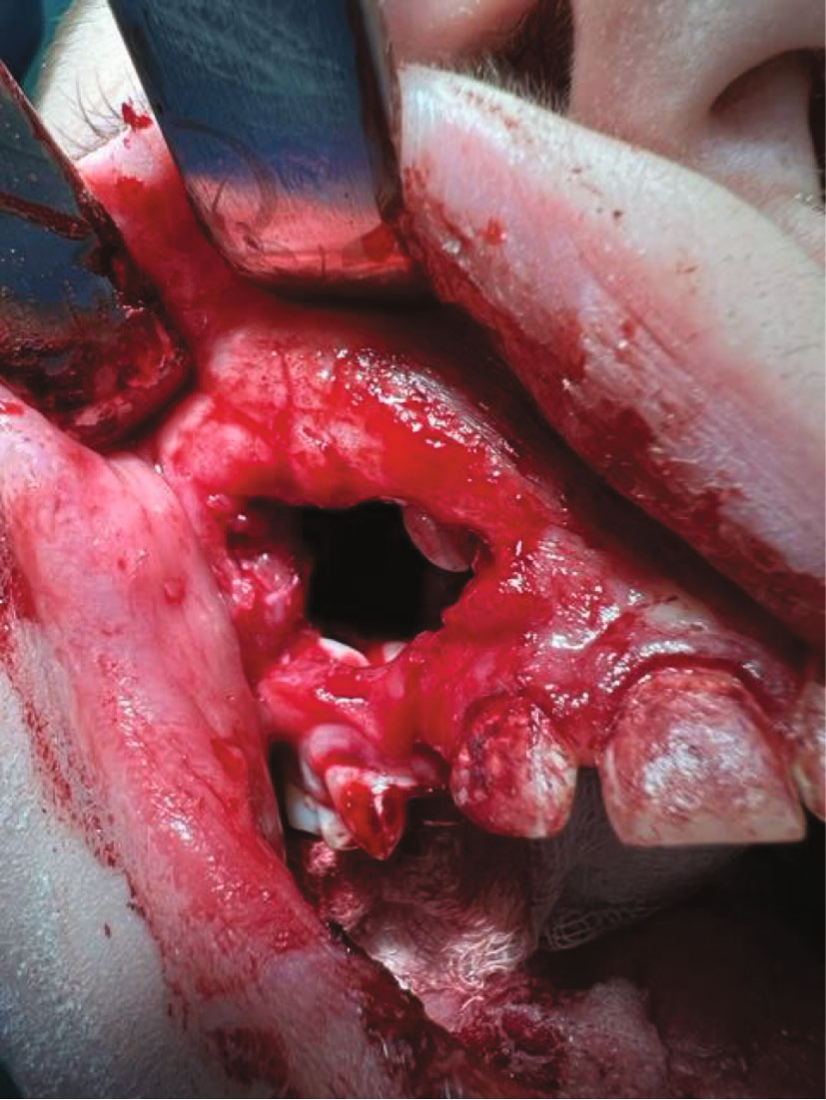
After incision and mucoperiosteal flap reflection, tooth 13 is visible. The anterior wall of maxillary sinus has been resorbed by the cyst.

**Figure 5:**
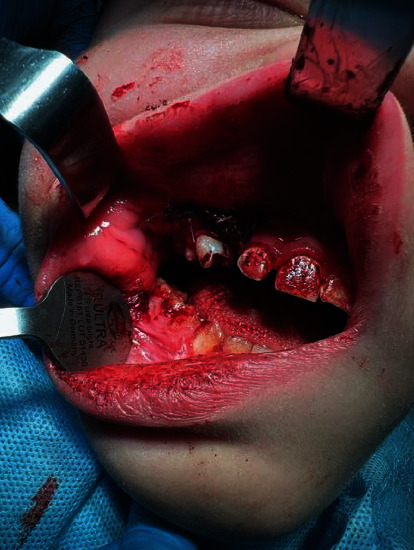
The wound closed with resorbable sutures. Depth of the vestibule is decreased due to the necessity of closing oroantral fistula.

Under general anesthesia (87.5 mg of propofol and 100 *μ*g of phentanyl) with monitoring of oxygen saturation and heart rate (by pulse oximeter attached to patient's finger), tooth 53 was removed and the fistula was excised (Figure 3). A full-thickness mucoperiosteal flap was designed through the excised fistula. The flap was reflected with anterior part of the cyst attached to it as the anterior wall of maxillary sinus was completely resorbed and tooth 13 became visible immediately (Figure 4). The next steps included separation of the cyst from the flap and enucleation of the entire lesion. We decided that the stabilization and position of tooth 13 are favorable to be used effectively in orthodontic treatment. The flap was repositioned with two layers of resorbable sutures: the first layer consisted of continuous suture (Novosyn 4/0) and the second of single interrupted sutures (Novosyn 3/0) to achieve better adaptation. The wound after tooth 53 extraction was sutured with a horizontal mattress (Novosyn 2/0). (Figure 5).

The patient regained consciousness in the recovery room and was discharged home. Postoperative antibiotic (150 mg of clindamycin every 8 hours) and analgesic (200 mg of ibuprofen every 6 hours) were prescribed.

**Figure 6:**
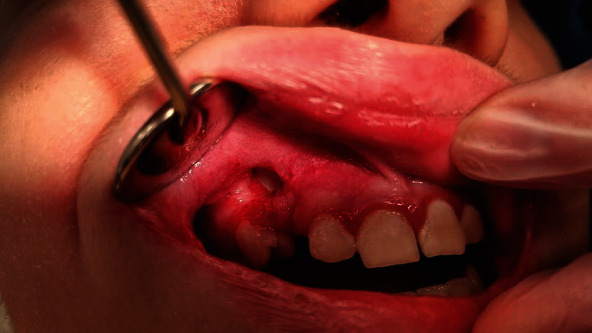
Intraoral view on operative area 2 months after surgery. Tooth 13 has started its eruption.

The follow-up appointments are as follows: first after 3 days, second after 2 weeks, and third after two months were scheduled. Proper healing of the wound and gradual reduction of the ailments were observed during first two visits. On the third appointment, the eruption of the tooth 13 was noticed, so the patient was referred for subsequent orthodontic treatment (Figure 6).

## 3. Discussion

Many articles describing dentigerous cysts have been published so far. The specific reason for this pathologic lesion remains unclear; however, numerous hypotheses are suggested. According to the “intrafollicular hypothesis,” a dentigerous cyst is a result of liquid accumulation between the external and internal surfaces of the epithelium. This amassing happens during the development of the crown. There is also the “enamel hypoplasia hypothesis” which proposes the formation of the cyst resulting from stellate reticulum degeneration. On the other hand, “Main's hypothesis” suggests that the cyst growth is a consequence of the hydrostatic tension applied by the affected tooth on the follicle, whose effect is the division of the affected crown from the encompassing follicle [2, 6]. According to histological evaluation, a dentigerous cyst can be described as inflammatory or noninflammatory. The former results from inflammation in a nonvital deciduous tooth, while the latter appears because of the pressure applied by an erupting tooth on an impacted follicle [2, 7].

Although dentigerous cysts usually occur as single lesions, cases of their bilateral formation have also been described. Although such situations are extremely rare, they have been reported in patients suffering from mucopolysaccharidosis, basal cell nevus syndrome (Gorlin syndrome), or cleidocranial dysplasia [8]. Among all the lesions that may be encountered in oral and facial areas, the following should be included in initial differential diagnosis due to their similar appearance in radiographic examination: radicular cyst, keratocystic and adenomatoid odontogenic tumors, unilocular ameloblastoma, and primordial cyst [3, 9, 10]. The three treatment methods of dentigerous cysts include enucleation, marsupialization, and decompression [1, 2, 4, 5, 11]. The decision which method should be used is based on the following criteria: cyst location and size, removal vs. preservation of unerupted tooth, pediatric patient's age and cooperation, and follow-up possibilities [2]. Marsupialization results in the formation of additional pocket in oral cavity from the cyst and is achieved by removal of its anterior wall. This method is considered conservative and is used when the preservation of the displaced teeth is desirable, especially in a young patient. It is also indicated in cases of huge cysts in mandible, whose enucleation could lead to bone fracture [12]. In the decompression method, a window in the cyst wall is created and a small acrylic device with a projectile is made to prevent the opening from closing. This results in a decrease of size of the cyst by internal pressure reduction and enables the enucleation to follow [13]. These two methods cannot be used in the area of maxillary sinuses for obvious reasons (e.g., chronic sinusitis).

In relation to cancer prevention, enucleation is the most favorable as the histopathological examination of the entire lesion can be performed and possible sites of neoplastic transformation can be detected. As an example, the following articles describe cancers arising from dentigerous cysts: intraosseous verrucous carcinoma [14] and primary intraosseous squamous cell carcinoma [15].

A method of treatment of massive dentigerous cysts involving the maxillary sinuses, not from the field of oral and maxillofacial surgery but otolaryngology, is the endoscopic-assisted transantral approach [16, 17]. The endonasal endoscopic approach by using various endoscopes and instruments is a minimally invasive surgery preserving physiological function while minimizing morbidity and preventing complications. Tooth and cyst that are close to the ostiomeatal complex can be managed through a fully endonasal endoscopic approach, preferably via a middle meatal antrostomy [17]. In our case, this method could not be used due to the lack of control over translocated canine and the presence of oroantral fistula.

## 4. Conclusions

Differentiation of infected cysts and intraoral abscesses still poses a serious problem for dentists who are not specialized in oral or maxillofacial surgery. Unfortunately, the inability to interpret radiographic examinations often results in misdiagnosis and inaccurate treatment. The necessity of more extensive education of dentists in the field of radiology should be emphasized.

## Data Availability

The references used to support the findings of this case report are listed in References.
